# Misbehavior During Penalty Kicks and Goalkeepers Holding the Ball Too Long as Trivial Offenses in Football

**DOI:** 10.3389/fpsyg.2019.00844

**Published:** 2019-04-18

**Authors:** Otto Kolbinger, Michael Stöckl

**Affiliations:** ^1^Chair of Performance Analysis and Sports Informatics, Department of Sport and Health Sciences, Technical University of Munich, Munich, Germany; ^2^Department of Sport Science, University of Vienna, Vienna, Austria

**Keywords:** trivial offenses, rule violations, football, penalty kicks, six-second-rule

## Abstract

Rule violations occur in every sport and the respective book of rules prescribes how match officials need to sanction them. However, there are some rule violations that are nearly never penalized, even if they are perceived by the match officials. A phenomenon that has been neglected in the scientific community so far, for which we want to introduce the term trivial offenses. This research focuses on two potential trivial offenses in football: rule violations regarding the six-seconds rule, the time a goalkeeper is allowed to control the ball with his hands, and rule violations during the performance of penalty kicks. The aim is to provide empirical proof of the existence of those trivial offenses and describe the respective patterns. For this purpose, two observation systems were constructed; one to investigate 45 games from the German Bundesliga with respect to the six-seconds rule and one to study rule violations during 618 penalty kicks from four European football leagues and one cup event. The following variables were collected: *Goalkeeper, MatchLocation, Minute* (representing the minute of the game), *PreviousAction, CurrentScore, Time* (representing the time the goalkeeper controlled the ball with his hands), and *Penalization* for the six-seconds study; *Responsibility for infringement, Decision of the referee*, and *Outcome* for the penalty study. Reliability tests showed almost perfect agreement for the data of both samples. On average, goalkeepers control the ball 6.0 s (SD:4.54) with their hands and the six-second rule was violated in 38.4% of the situations, none of which was penalized. This duration was significantly influenced by *CurrentScore* (*p* < 0.001), which indicates a tactical abuse of this situation. None of the investigated penalty kicks was conducted without a rule violation either. In most incidents (96.3%) outfield players from both teams as well as the goalkeeper commit offenses. The umpire only judges 2.8% of these incidents correctly, most of them by approving the scored goal. In total, this research proves the existence of trivial offenses in football and shows how methods and tools of performance analysis can serve to investigate and even solve this issue.

## Introduction

The International Football Association Board (hereinafter referred to as IFAB) claims in their current version of the official book of rules, pretentiously called *Laws of the game*, “[that] the same Laws apply in every match in every confederation, country, town and village throughout the world is a considerable strength which must be preserved” and further that “[the] integrity of the Laws, and the referees who apply them, must always be protected and respected” (IFAB, 2018, p. 11). These statements might be rhetorically overflourished, but they are in line with views of sport theorists, which describe rules as a substantial part of the answer to the metaphysical question “what is (a) sport?” (Reid, 2012). Suits (1988) describes how rules are the underlying reason that specific skills are developed, for example to control the football with all parts of the body except the upper extremities, as the deliberate use of these is prohibited by the rules. Of course, rules can fulfill further purposes, for example to ensure player’s safety, but all contribute to the matter of defining a (game) sport. Besides this, sport philosophers also believe in the concept of informal agreements about the way a sport should be performed (D’Agostino, 1995). Such so-called unwritten rules cover various aspects which also can include an agreement of the acceptance of minor rule violations.

It is a well-accepted public perception in many sports that there are frequent minor rule violations, which are not punished by the umpires or referees (both terms are used interchangeably throughout this manuscript, based on the specific terminology of the respective sports). A specific part of unwritten rules for which we wish to introduce the term *trivial offenses* in sport. However, whereas there is already a solid research base for some areas of refereeing behavior, like the existence of different sorts of bias (see the well-structured review of Dohmen and Sauermann, 2015) and how this bias is influenced by different external factors (crowd noise: Unkelbach and Memmert, 2010; crowd proximity: Scoppa, 2008), just two trivial offenses have been subjected to scientific publications so far. Based on an incident at the 2009 US Open Women’s semi-finals, Serena Williams threatened a line judge after a punished foot fault, two legal theorists discussed foot-faults as an issue of temporal variance: Should foot-faults only be called in less important stages of a match or all the time? Whereas Standen (2013) argued that offenses need to be punished at all times, Berman (2011) saw circumstances in which a no-call could be more beneficial for the sake of a sport. The only empirical study with an emphasis on trivial offenses also included the violation of a restriction that should ensure a specific distance. In an evaluation of the vanishing spray in football, Kolbinger and Link (2016) found that, even after the introduction of this officiating aid, the minimum distance rule was violated frequently but not punished a single time in their sample. This work of Kolbinger and Link (2016) is so far the only published research that uses methods and tools of performance analysis to investigate the phenomenon of trivial offenses in sport. Therefore, the aim of this study is to investigate two further issues of trivial offenses in football: The rule applications during penalty kicks (hereinafter referred to as *penalty study*) and the enforcement of the rule that prohibits the goalkeepers from controlling the ball with their hands for more than six seconds (hereinafter referred to as *six-seconds study*). For both, there is the common belief that the respective situations are often not conducted in accordance with the prevailing rules.

Watching penalty kicks, one often has the impression that at least one player is breaking the rules and sometimes even more than one. Regarding penalty shootouts, there are non-scientific analyses based on small sample sizes that seem to underpin this common belief. For instance, a German football magazine stated that 16 out of 20 penalty kicks during two round four matches at the World Cup 2018 in Russia were not performed correctly (Kicker, 2018). However, we were not able to find scientific research that investigated rule violations with respect to penalty kicks in football. This is quite surprising, as up to 10% of all goals result from penalty kicks (Yiannakos and Armatas, 2006; Wright et al., 2011; Michailidis et al., 2013). To overcome this lack of research, the aim of the *penalty study* is to investigate the conducting of penalty kicks in soccer in four professional European leagues and one cup event. Of particular interest is if and in which way penalties are conducted irregularly and what the referee’s decision was in the respective situation. However, only penalty kicks that were not a part of penalty shootouts were considered.

Concerning the *six-seconds study*, there is no scientific literature either. As it often seems that goalkeepers of teams that are in the lead holding on to the ball for more than six seconds and delaying the game is a widespread – and promising (Siegle and Prüßner, 2013) – practice in football, we want to investigate the influence of the score and the elapsed game time on the ball-in-hand time. Further, we look for additional variables that could influence this duration, like match location, game context and individual characteristics. Based on the findings of both studies, we furthermore would like to demonstrate how methods and tools of performance analysis can be used to identify trivial offenses and support associations to solve the respective issues.

## Materials and Methods

### Data Collection

For the *penalty study*, an experienced operator extracted the data from video footage of football matches provided by Sportradar. Matches from four European leagues (Austrian Bundesliga, German Bundesliga, Serie A, and Premier League) and one cup event (DFB-Pokal, the German cup event) from two complete seasons (2015/16, 2016/17) and the beginning of the 2017/18 season were considered. Parameters for each penalty kick that occurred during one of these matches were collected using an observation system that will be introduced in the following paragraphs. In total, 618 were investigated (cf. Table 1).

**Table 1:** Distribution of the investigated penalty kicks with regard to the leagues and the cup event.

	Austrian Bundesliga	German Bundesliga	Serie A	Premier League	DFB-Pokal	Total
2015/16	31	60	56	40	19	38
2016/17	41	98	127	92	17	374
2017/18	6	6	11	3	12	206
Total	78	166	194	135	48	618


The information on the *penalty study* was collected using a systematic observation system considering the Laws of the Game. The FIFA book of rules states the following guidelines that describe a penalty kick taken in accordance with the rules (IFAB, 2018):

- Regarding all players of both teams (excluding the player that performs the penalty kick and the respective goalkeeper): Before a penalty is taken, they have to be outside the penalty area, but within the field of play and behind the penalty mark, at least 10 yards (9.15 m). Further, these players are not allowed to enter the penalty area before the kick is taken.
- It must be obvious for the goalkeeper who takes the penalty kick. Further, the goalkeeper has to stand on the goal line between the goal posts until the penalty kick taker touches the ball.
- The penalty kick taker can only conduct the penalty kick after the referee blows his/her whistle indicating that the penalty kick can be conducted. During the run-up the penalty kick taker can make feinting moves until he/she touches the ball. The ball must be kicked forward toward the goal.

In case of rule violations, the FIFA book of rules instructs the referee how to decide who is responsible for the infringement and the outcome of the penalty kick (cf. Table 2).

**Table 2:** Overview on how the referee has to decide considering the outcome of the penalty kick.

Responsibility for rule infringement	Goal	No goal
Player(s) of attacking team	Rekick	Indirect free kick
Player(s) of defending team	Goal	Rekick
Both	Rekick	Rekick


The parameters gathered for this part of the study were the following:

- *Country*: Austria, England, Germany, and Italy
- *Event*: League, Cup
- *Year*: 2015/16, 2016/17, 2017/18
- *Responsibility for infringement* (*Rfi*):
  • Goalkeeper, player(s) of attacking team, player(s) of defending team
  • Player(s) of both teams
  • Combinations of the goalkeeper and player(s) of one team or both teams
  • No infringement
- *Decision of the referee* (*Df*): Binary, recognizing an infringement or not
- *Outcome*: Goal, saved by goalkeeper, or shot misses goal

The data for the *six-seconds study* was recorded manually by an expert (sport science student in his final semester) using the broadcast footage of 45 games from the German Bundesliga (random sample). Each team had a minimum occurrence of four games and a maximum of six. Using a self-designed systematic observation system, the following attributes were collected for each of the 458 ball-in-hand incidents: *Goalkeeper, MatchLocation, Minute* (representing the minute of the game), *PreviousAction, CurrentScore, Time* (representing the time the goalkeeper controlled the ball with his hands) and *Penalization*. The levels of the attribute are listed in Table 3. *Time* was measured using an ordinary stopwatch.

**Table 3:** Observational system used for the six-seconds study.

Attribute	Attribute levels and/or operationalizations
*Goalkeeper*	Name of the goalkeeper
*MatchLocation*	*Home*: Goalkeeper playing for the hosting team *Away*: Goalkeeper playing for the visiting team
*Minute*	Integer between 1 and 90. Extra time was assigned to the values 45 and 90, respectively
*PreviousAction*	*Shot*: Opponent performed a shot on goal as last action before the goalkeeper gained control of the ball *Cross*: Goalkeeper intercepts a cross *Pass*: Teammate passing the ball to the goalkeeper in a way which allows the goalkeeper to control the ball with his hands *Other*: Otherwise
*CurrentScore*	Integer that illustrates the goal difference from the goalkeeper’s perspective (positive value means winning, negative value means losing and zero means drawing)
*Time*	Time between the moment the goalkeeper starts to control the ball with his hands according to rule 12 of the official “Laws of the Game” and when it finally leaves his hands. Measured in seconds (one decimal)
*Penalization*	*True*: Referee award an indirect free kick to the other team *False*: Otherwise


### Reliability

The reliability of the *penalty study* was tested using an intra-rater reliability test. For this, the operator collected data from 100 randomly chosen penalty kicks for a second time, one month after the first observation. The reliability of the data was assessed regarding the actual agreement and Cohen’s kappa. For *Country, Year, Event, Df*, and *Outcome* there was perfect agreement (100%), which means Cohen’s kappa equals 1. Regarding *Rfi* the actual agreement was 99% and κ = 0.944.

The inter-rater agreement for the *six-seconds study* was performed for 103 incidents over nine matches, using a research assistant with six years of experience in game observation as the second independent observer. There was a perfect agreement (Cohen’s kappa equals one) for *Goalkeeper, MatchLocation*, and *Minute* (which was treated as nominal variable for this purpose), *PreviousAction* and *CurrentScore*. It was not possible to calculate Cohen’s kappa for *Penalization*, as the same level occurred in all situations for both observers (which equals an agreement percentage of one). For *Time* we calculated the linear correlation coefficient, which was 0.99, and some descriptive statistics to further describe the agreement. Mean difference between the two observers was 0.01 s and the mean absolute difference 0.09 s with an absolute maximum difference of 0.5 s. In total, there is almost perfect agreement for both studies (Landis and Koch, 1977).

### Statistical Analysis

The descriptive and inferential analysis for the *penalty study* were performed using SPSS (IBM Corp. Released 2016. IBM SPSS Statistics for Windows, Version 24.0. Armonk, NY, United States: IBM Corp.). Statistical differences were determined using the chi-square test and, where necessary, using its non-parametrical equivalent the Fisher’s exact test (based on Monte Carlo simulation). Effect sizes were determined using Cramer’s V and interpreted based on the limits suggested by Cohen (1988).

For the *six-seconds study*, descriptive statistics were used to show the prevalence of rule violations and the frequency of respective sanctions by the referee. Further, we ran regression analysis to describe how the collected variables (*Goalkeeper, MatchLocation, Minute, PreviousAction*, and *CurrentScore*) influence the ball-in-hand time (*Time*). Two of those variables were polytomous: *Goalkeeper* and *PreviousAction*. Both were included without further processing in Model 1, which means that both were transformed into dummy variables. In more detail, for *Goalkeeper* this led to 23 dummy variables (as 24 goalkeepers appeared in our sample) that equal one if the respective goalkeeper was involved and zero if not. For *PreviousAction*, we had to include three dummy variables (as we pooled the previous actions into four categories) that equal one if the ball-in-hand incident was initiated by a certain action and zero if not.

$$Time_{i}=\beta_{0}+\beta_{1}Goalkeeper_{i}+\beta_{2}MatchLocation_{i}+\beta_{3}Minute_{i}+\beta_{4}PreviousAction_{i}+\beta_{5}CurrentScore_{i}+\varepsilon_{i}$$

To improve comprehensibility, we performed a second regression model (Model 2), for which we transformed the two polytomous variables. *Goalkeeper* was substituted by *GK_Mean*, which displays the average ball-in-hand time for each goalkeeper. This transformation does not interfere with the objectives of this study, as the aim is not to gain knowledge about an increase or decrease in the ball-in-hand time that is affected by a certain goalkeeper. Instead, we wanted to show how much of the respective variance is explained by interindividual differences. Further, based on the descriptive results, *PreviousAction* was transformed into a dichotomous variable for Model 2, equaling one if *PreviousAction* was classified as *Shot* or *Cross*.

$$Time_{i}=\beta_{0}+\beta_{1}GK\_Mean_{i}+\beta_{2}MatchLocation_{i}+\beta_{3}Minute_{i}+\beta_{4}PreviousAction_{i}+\beta_{5}CurrentScore_{i}+\varepsilon_{i}$$

To clarify the appropriateness of the data processing for the second model, which should be used for the interpretation of the results, the two models were compared in order to check for differences in goodness of fit.

None of the included metric variables was z-transformed. First, we think it is more beneficial to describe the increase or decrease of the dependent variable *Time* in seconds for each predictor, not standard deviations. Vice versa, the same applies for the predicting variables *Minute* and *CurrentScore*. The severity of multicollinearity was measured using the variance inflation factor (VIF). The data processing and the statistical analysis for the *six-seconds study* were conducted in R (R Development Core Team, 2015).

## Results

### Penalty Study

Descriptive analyses (Table 4) of the investigated penalty kicks reveal that there was no penalty that was conducted according to the prevailing football rules. In most of these cases (96.3%), even players of both teams (including the goalkeeper) misbehaved. This varied slightly from league to league (*p* < 0.001; Cramer’s *V* = 0.13). In the German Bundesliga (93.9%), and in particular in the Austrian Bundesliga (88.5%) there were fewer incidents in which players of both teams (including the goalkeeper) broke the rules. However, this proportion was much higher in the English Premier League (99.3%) and the Italian Serie A (98.5%). No statistical difference can be found between the cup competition and the leagues (*p* = 0.809).

**Table 4:** Descriptive results are presented with respect to infringements, who is responsible for the infringements, and how many of the respective cases were correctly judged by the umpire.

Infringement	Infringement by	% (n)	Referee’s decision correct in % (n)
**No**		0.0% (*n* = 0)	
**Yes**	Total	100.0% (*n* = 618)	2.8% (*n* = 17)
	Attackers	–	–
	Defenders	0.3% (*n* = 2)	100.0% (*n* = 2)
	Both	1.0% (*n* = 6)	0.0% (*n* = 0)
	Goalkeeper	–	–
	Goalkeeper + Attackers	–	–
	Goalkeeper + Defenders	2.4% (*n* = 15)	80.0% (*n* = 12)
	Goalkeeper + Defenders + Attackers	96.3% (*n* = 595)	0.5% (*n* = 3)


In total, the referees correctly judged 2.8% (*n* = 17) of the infringements. In most of these incidents (82.4%; *n* = 14) the referee judged correctly by not actively interrupting the game because the penalty kick resulted in a goal and the players of the attacking team behaved according to the football rules. In three cases, the referee actively interrupted the game and made the players repeat the penalty kick due to the misbehavior of outfield players from both teams as well as the goalkeeper.

### Six-Seconds Study

**FIGURE 1:**
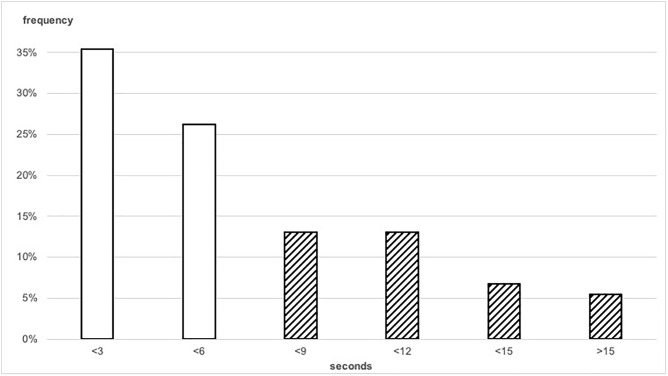
Histogram for the ball-in-hand times, pooled into three-second bins. Crosshatched bars illustrate groups that exceed the six second maximum.

The goalkeepers in our sample controlled the ball with their hands for an average time of 6.0 s (SD:4.54 s), which equals the permitted six seconds mark. As illustrated in Figure 1, the respective rule was violated in 38.4% of the observed ball-in-hand situations. For 12.2% of incidents the time limit was exceeded for more than six seconds, the maximum value was 20.1 s. None of the rule violations were penalized.

Table 5 shows both models, as well as additional information for the included variables in column two. For the metric predictor variables, we calculated the correlations with the dependent variable *Time*, which were significant for *GK_Mean* (*r* = 0.33, *t* = 7.38, *p* < 0.001) and *CurrentScore* (*r* = 0.23, *t* = 5.09, *p* < 0.001). In more detail, *Time* is on average under six seconds for any score for trailing teams and above six seconds for teams in the lead. For the nominal variables, we calculated descriptive statistics and compared the respective attribute levels. As we show in column two of Table 5, if the *PreviousAction* was labeled as shot or cross, *Time* was significantly higher compared to the other levels.

**Table 5:** Correlation coefficient with the inter-point time for metric variables and descriptive statistics of the nominal variables included in the summarized Models 1 and 2.

	Correlation with *Time*	Model 1	Model 2
		
	*R*^2^ (*t*-value)	Coefficients (*t*-value) *VIF*	
*GK_Mean*	0.107^∗∗∗^		0.961^∗∗∗^
	(7.38)		(7.57)
			*1.01*
*Minute*	0.001	0.001	0.001
	(0.66)	(0.17)	(0.15)
		*1.04*	*1.01*
*CurrentScore*	0.054^∗∗∗^	1.01^∗∗∗^	0.775^∗∗∗^
	(5.09)	(5.95)	(5.10)
		*1.27*	*1.02*

	**Time in seconds Mean ± SD**		

*PreviousAction < Shot >*	7.78^cd^ ± 4.84	2.64^∗∗∗^	2.345^∗∗∗^


		(5.22)	(6.20)
		*1.03*	*1.02*
*PreviousAction < Cross >*	7.28^cd^ ± 4.97	2.43^∗∗∗^	


		(4.87)	
		*1.03*	
*PreviousAction < Pass >*	4.64^ab^ ± 4.15	-0.121	*base group*


		(-0.19)	
		*1.03*	
*PreviousAction < Other >*	4.76^ab^ ± 3.73	*base group*	
*MatchLocation < Home >*	5.55 ± 4.59	-1.31^∗∗^	-0.853^∗^


		(-3.16)	(-2.25)
		*1.19*	*1.01*
*MatchLocation < Away >*	6.27 ± 4.49	*base group*	*base group*
*Goalkeeper* Dummies		Included	excluded
Intercept		2.79^∗^	-0.497^∗∗∗^
		(2.53)	(-0.59)

		**Goodness of fit**	

*F* (df, n)		5.24^∗∗∗^	28.0^∗∗∗^
		(29,428)	(5,452)
*R*^2^		0.262	0.236
Adjusted *R*^2^		0.212	0.228


*^∗^ significant on 0.05 level; ^∗∗^ significant on 0.01 level; ^∗∗∗^ significant on 0.001 level. ^a^ significantly different from <Shot> (on 0.05 level); ^b^ significantly different from <Cross> (on 0.05 level); ^c^ significantly different from <Pass> (on 0.05 level); ^d^ significantly different from <Other> (on 0.05 level).*

As Model 2 only showed a minor decrease of the *R*^2^_adj_ and no significant difference compared to Model 1, we use the results of Model 2 to describe and interpret the influence of the predictor variables. *Time* increases by 2.34 s if the previous action was a shot or cross and decreases by 0.85 s if the goalkeeper is playing for the home team. *Minute* does not show a significant independent influence on the ball-in-hand time. Concerning individual influence, an additional second at *GK_Mean* increases *Time* by 0.96 s. The coefficient for the current score difference shows a highly significant influence as well (*t* = 5.10, *p* < 0.001), with an average increase of 0.78 s for each positive increase in the goal difference.

## Discussion

There is a common belief among various stakeholders from causal fans to sport theorists that some sports have unwritten rules, which includes the intentional mishandling of some rules. In this paper we could provide empirical proof supporting this common believe for two such trivial offenses: various misbehaviours during the performance of penalty kicks and violations of the maximum time limit a goalkeeper can have control of the ball with his hands.

Regarding the *penalty study*, this research shows that all investigated penalty kicks were not conducted as the book of rules prescribes. Misbehavior occurs in all investigated European football leagues. During each penalty kick at least one player commits a rule violation. Interestingly, in most incidents none of the parties involved behaves correctly. However, it is not too surprising that players try to stretch the rules as far as possible in order to profit from it. In most incidents, the fact that the players misbehave might be a sequence of reactions started by just one player moving too early and, concurrently, other players following to compensate for the potential disadvantage. For instance, if an attacker enters the penalty area first in order to get a potential rebound, a defender is probably going to follow in order to compensate for the disadvantage.

Nearly all refereeing decisions were wrong. There are three different scenes a referee and his assistants have to monitor: The penalty kick taker, the goal line (observing the goalkeeper), and the eighteen-yard line (observing the rest of the players). On the one hand, there is one match official focusing on each scene. This actually seems to be an easy task. On the other hand, all match officials need to recognize when the penalty kick taker touches the ball and, at the same time, judge whether another player commits a rule violation. Firstly, this is impossible and secondly, sometimes players only slightly violate the rules in such a way that a human being might not be able to perceive it in real time. Unfortunately, we did not collect information about the amount of time a player misbehaved early, or how far a player entered the penalty area, or how far the goalkeeper left the goal line. This kind of information should be collected in a future study to help in answering this question. In the course of this research, there was the opportunity to stop the video and check each single frame to identify rule violations. As the video assistant referee has been introduced in several leagues today, this opportunity also exists for match officials. However, it seems that the misbehavior during penalty kicks is still there, but this should be part of further research as the data of the *penalty study* was recorded before the introduction of the video assistant.

Similar to Berman’s (2011) arguments in his tennis research, referees could misjudge misbehavior during penalty kicks to keep the flow of the game, which might be an even bigger issue in an invasion game like football. Further, a team gets a penalty kick to compensate for a disadvantage. Having a penalty kick should be an advantage for a team and, therefore, it should profit from this situation. Since in 80% of the cases a penalty kick results in a goal (Kropp and Trapp, 1999; Bar-Eli et al., 2007; Wright et al., 2011), referees might render the misbehavior as no longer relevant in these situations.

Regarding the *six-seconds study*, we found that in almost 40% of the observed ball-in-hand incidents the goalkeepers held on to the ball for more than six seconds. For almost one third of these violations the ball was not released until the permitted time interval had elapsed twice over. As expected we found indications that the ball-in-hand situations, in which the goalkeeper cannot be challenged by an opponent, is used to delay the game. If the goalkeeper’s team has the lead, the average ball-in-hand time is 7.07 s, compared to 4.23 s when the team is trailing. The amount of rule violations increases from 25.4 to 46.2%. A strategy that is not surprising, considering the systematic underestimation of the additional time (Siegle and Prüßner, 2013). In contrast, the elapsed game time influenced neither the dependent variable nor the share of rule violations.

*PreviousAction* showed the highest correlation with the ball-in-hand time among our context variables. If the goalkeeper had to save a shot or intercept a cross, the ball-in-hand time increased by 2.7 s and the number of situations in which the six-seconds maximum is exceeded increased by 22%. Further, significant parts of the variance in time were affected by the variance of the goalkeepers and by the match location (whether the match was played at home or away). Further, we want to note that our model was only able to explain about 23% of the total variance of the dependent variable. Thus, there are further variables that have an impact on the ball-in-hand time. One that probably has an impact on the time the goalkeeper controls the ball with his hand is the spatial distribution of his teammates and opponents. The goalkeeper could perceive a potential tactical advantage by releasing the ball quickly or, alternatively, conclude that it would be a disadvantage to speed up the game in a particular situation. Another condition that could affect the ball is the status of the involved teams. Some teams might be satisfied with a tie, for example underdogs or teams that have suffered a red card. It needs to be mentioned that, in contrast to the *penalty study*, the results are limited to patterns in the German Bundesliga, as we were not able to collect this data for other European leagues.

In general, for both situations covered in this paper, the referees are more or less expected to not penalize the respective rule violations, which makes these situations a typical example of unwritten rules (D’Agostino, 1995). However, this kind of unwritten rules, for which we introduced the term *trivial offenses*, can cause serious problems, as Standen (2013) and Berman (2011) show in their respective papers about temporal variance. Especially, in cases in which erratic rule applications interfere with the main objective of a rule regarding the related athletic skills. The goal in soccer is to score more goals than the opponent in a defined amount of time. Controlling the ball with the hand for a longer time than the permitted six seconds (again: the goalkeeper cannot be challenged when he controls the ball with his hands) takes away a share of this time in which the opponent can try to tie or win the game based on their athletic skills. For penalties there can be a huge shift in the preconditions set by these skills as well, especially if the goalkeeper shortens the distance to the penalty mark.

Accepting minor rule violations, even if it might increase the entertainment value, would counteract IFAB’s goal of protecting the integrity of the game. Thus, we claim that the IFAB needs to step in and either prompt the referees to enforce the respective rules according to the Laws of the Game or change the respective rules. For example, the IFAB could prolong the time permitted for goalkeepers to hold on to the ball to eight seconds after shots or crosses or change the penalizations for rule violations during penalty kicks. The latter would make sense if the current penalization is seen as too much of a disadvantage for the offensive team. This would need further evaluations, including surveys of the opinions of various stakeholders. To sum up, the IFAB needs to change the rules or the refereeing behavior if it wants to protect the integrity of its rules. Using tools and methods of performance analysis, this study not only provided empirical proof of the existence of the trivial offenses, but also additional information about the respective situations which can contribute to the process of changing the way these situations are umpired.

## Author Contributions

MS performed the penalty study. OK performed the six-seconds study and provided the theoretical framework for trivial offenses. Both authors contributed to the manuscript, with emphasis on their respective studies.

## Conflict of Interest Statement

The authors declare that the research was conducted in the absence of any commercial or financial relationships that could be construed as a potential conflict of interest.
